# External Supervision, Face Consciousness, and Pesticide Safety Use: Evidence from Sichuan Province, China

**DOI:** 10.3390/ijerph19127013

**Published:** 2022-06-08

**Authors:** Dakuan Qiao, Lei Luo, Xingqiang Zheng, Xinhong Fu

**Affiliations:** School of Management, Sichuan Agricultural University, Chengdu 611130, China; 2020309003@stu.sicau.edu.cn (D.Q.); luolei@stu.sicau.edu.cn (L.L.); zxq2096115807@163.com (X.Z.)

**Keywords:** external supervision, face consciousness, pesticide safety use, intergenerational differences, binary logit, IV-2SLS

## Abstract

Clarifying the factors influencing the safe use of pesticide is essential for scientific decision making to effectively manage pesticide use and promote sustainable agroecological development. The study aims to explore the factors influencing farmers’ safe use of pesticides from the perspectives of external supervision and face consciousness. Using survey data covering 534 farm households in Sichuan province, this study empirically analyzes the influence mechanisms of external supervision, face consciousness, and their interaction terms on farmers’ safe use of pesticides by employing the binary logit and IV-2SLS model, and further reveals their intergenerational differences on this basis. The results show that external supervision and positive face consciousness have significantly positive effects on pesticide safety use by farmers; market supervision and ability-type face, respectively, play the biggest role among them. Furthermore, there is an interaction effect between external supervision and face consciousness with respect to pesticide safety use. Farmers with different generation farms are influenced differently by external supervision and face consciousness. The behaviors of the new generation farmers to safely use pesticide are principally influenced by government supervision, market supervision, and ability-type face; in contrast, the ones of the older generation farmers are mainly influenced by market supervision, organization supervision, and relationship-type face.

## 1. Introduction

Pesticides have been widely used in agriculture in order to control pests and boost crop yields [1], and they are now a key input of modern agriculture globally [2]. Over the past few years, China had become the largest pesticide user in the world, with nearly 1.392 million tons of pesticide consumption in 2019 [3], and applied 2.5 times more pesticides per hectare than the global average. The intensity of pesticide used was 10.31 kg/hm^2^, which is much higher than the internationally accepted upper limit of 7.5 kg/hm^2^ for the safe use of pesticides [4], and, to solve the environmental pollution problem caused by the overuse of pesticides in China and achieve a dynamic balance between agricultural pest control and environmental protection, it is necessary to explore new paths to safely use pesticides.

Unsafe pesticide use practices will cause a series of adverse consequences. Irrational and unscientific dispensing before using pesticides makes the concentration of pesticides higher than the prescribed standard, which directly harms the agricultural environment and the safety of agro-products [5]. During use, most farmers overuse chemical pesticides to cope with pest and disease hazards, resulting in pesticide residues in the fields and water bodies, thus polluting the soil, atmosphere, and water environment to varying degrees [4]. More worryingly, farmers neglect to take safety precautions to reduce the risk of poisoning during the use of pesticides, which seriously threatens their own health [6]. Further, the safety interval of pesticides is an important parameter affecting the amount of pesticide residues, a study has shown that some farmers do not wait for metabolic decomposition after applying pesticides to pick fruit, resulting in excessive pesticide residues and metabolites in agro-products [7]. According to a survey, 62% of farmers directly throw the used waste packaging into the field or water bodies after application, and more than 3.2 billion pesticide waste boxes are discarded randomly every year, and 2–5% of pesticide residues in the boxes may be released into the surrounding environment with rainfall or irrigation [3], causing irreversible pollution of the agroecosystem.

To address the issues of unsafe pesticide use, scholars have conducted a wealth of research, which mainly focuses on the following aspects, Firstly, the endowment characteristics of farm households. Most scholars have obtained consistent findings based on different research materials and methodologies; they believe that farmers’ age, educational attainment, risk preference, household size, farming size and experience, and other personal, household, and production characteristics are inextricably linked to the safe use of pesticides [8,9,10,11]. Secondly, the drivers influencing farmers’ safe pesticide use. From an internal perspective, most studies have empirically explored the influence of farmers’ risk perceptions, subjective norms, and their perceived values on safe pesticide use based on the farmers’ behavioral theory [12], planned behavior theory [13], and health belief theory [14]. From an external viewpoint, numerous scholars find that external factors, such as information access [15], external risk [12], technological environment [16], differentiated policies [17], skills training [18], and the economic environment, also play an increasing role in influencing pesticide safety use [18,19]. However, significant differences between farmers’ perceptions of safety behavior and actual behavior status [20] make the factors influencing farmers’ safety production gradually become more abundant; for example, Guo et al. (2021) conducted a study about rice farmers’ safe production and found that farmers’ behavior resulted from a combination of internal and external factors. Thirdly, the outcome variables of pesticide use behavior. The uncontrolled use of chemical pesticides poses serious environmental hazards and affects the stable supply and long-term use of agricultural labor quality [21]; in contrast, regulated pesticide use behaviors can significantly increase the income level of farm households [22].

Existing research findings contribute meaningful literature support to the paper’s analysis, but it is easy to find through combing the literature that existing studies mostly discuss the impact of certain internal or external factors on farmers’ safe production separately, and few studies consider internal and external factors in unison, yet farmers’ behaviors result largely from both internal and external factors [23]. Among the internal factors, one study has found that face consciousness has long been rooted in Chinese society [24] and profoundly influences farmers’ behavior. Through face, it is possible to establish a highly trusting “one’s own” relationship, and the very notion of face constitutes the purpose of action [25]. Among the external factors, the external supervision is an essential component and has been the focus of studies. Meanwhile, as an important component of the system, it is a formal constraint that has been applied in various areas of social work and supremely influenced on the effectiveness of individual self-regulation [26,27].

Based on the above analysis, this paper will make theoretical and practical contributions to related research from the following aspects. Firstly, we attempt to construct an analytical framework that includes face consciousness, external supervision, and pesticide safety use to explore the impact of these two factors on pesticide safety use. Secondly, this study further examines the interactive effects of these two factors on farmers’ safe pesticide use. Finally, intergenerational grouping of farmers is conducted to explore the effects of face consciousness and external supervision on safe pesticide use among different generations of farmers.

## 2. Research Hypothesis

### 2.1. Effects of External Supervision on Pesticide Safety Use

Mayer and Smith (1982) were the first to propose the external supervision hypothesis upon the study of corporate governance. As the research progressed, the study of the effect of external supervision on individual behavior gradually achieved application and development in the fields of business management [28], administration [29], social work [26], and rural environmental governance [3]. However, scholars perceived and defined external supervision differently, often adapting it appropriately to their research topics. For example, Tang (2011) proposed that the force of power supervision came from outside the power exerciser based on the study of administrative management; according to Beddoe (2012), external supervision is defined as supervision between practitioners and supervisors of nonidentical organizations. As we can find, the definition of “supervisory subject and supervisory object belong to a non-subordinate organizational system” is commonly accepted by academics. Given this, this study defines external supervision as “the supervision of farmers’ behavior by institutions or groups other than individual farmers”. Some scholars have found that government, market, and organization are three crucial subjects of external supervision, the joint regulatory role of the three exerts positive effects on farmers’ pesticide safety use and ensures agrifood quality and safety [30]. More specifically, market instruments, being an efficient and flexible environmental governance tool, are closely related to farmers’ pro-environmental behaviors due to its economic rationality [31,32], and the implementation of a minimum quality standard access system can stimulate the market selection mechanisms to increase opportunistic costs, reduce the supply of low-end products, and improve the average price of high-quality products [33], thereby guiding farmers to safe production. However, market mechanisms are not omnipotent; where the market fails, the government’s function, nature, and public goods character of the environment as a representative of the public interest dictate that its environmental oversight serves a crucial role in promoting the adoption of environmentally friendly practices by farmers [11]. Additionally, some scholars have emphasized that peasant co-operatives, as complements to market and government regulation, would promote farmers’ practice of safe production and ensure ecological and food safety by reinforcing rules and sending signals [34].

### 2.2. Effects of Face Consciousness on Pesticide Safety Use

Face consciousness belongs to the personality traits of individuals. As early as 1955, Goffman stated that “face is a form of social respect and identity, a positive social value that is strongly asserted by an individual in a particular social interaction” [35]. Subsequently, Brown and Levinson (1987) defined it as the public self-image that individuals hoped others to recognize [36]. According to politeness theory, face should be a universal concept, only that its definition varied in different cultural contexts. However, for China, Hu (2005) was the first to interpret and distinguish face [37]. Subsequently, based on the context of traditional Chinese Confucian culture, some scholars had synthesized previous studies and found that Chinese face was a multidimensional concept involving at least three elements of ability, morality, and relationship, namely ability-type face, morality-type face, and relationship-type face [38]. Most of the current research involving the impact of face on individuals’ green behavior has focused only on consumption domain, and few have explored its impact on green production in agriculture, especially pesticide safety use. This research argues that individuals with strong face recognition demand higher social value gains, such as reputation and social support. The social benefits generated by green consumption could satisfy this demand to some extent, thus motivating consumers to consume green [39,40,41]. Whilst green production has been gradually entering the mainstream concept advocated by society, safe pesticide use has become a pro-environmental practice. Damage to the collective environment of the village will affect the reputation and image of the farmer among the public; in contrast, practicing safe pesticide use to protect the environment and food safety will project favorable public image to society and improve their social status among surrounding groups; therefore, farmers with intense face mentality would probably rather earn the recognition and respect of others through safe pesticide use.

### 2.3. An Analytical Framework for the Impact of External Supervision and Face Consciousness on Safe Pesticide Use by Farmers

Farmers’ willingness towards safe pesticide use is simultaneously influenced by both face consciousness and external supervision. In general, external supervision could lead to a specific “dominant response” (The “dominant response reinforcement theory” was proposed by Zajonc, R.B. in 1965 to explain the phenomenon of social facilitation and social disruption) by subjects, thus manifesting as an internal drive [42], while face consciousness acting as behavioral norms for people tended to enhance this force, especially with public affairs and situations [43]. In the case of pesticide safety use, ignoring face consciousness, external supervision can create impacts on pesticide use behaviors and increase the probability of the behaviors, i.e., those who use pesticides illegally will be criticized and punished once they are discovered, which will cause them to incur losses to their financial interests and public image, and will raise their probability of applying pesticides safely. In addition, if face consciousness works, this punishment can damage the reputation and self-image of farmers in their village collectives and other social places. Influenced by traditional face thinking in rural society, farmers will engage in safe application, thus intensifying the restraining effect of external supervision. From another perspective, farmers with powerful face tend to produce safely, and the involvement of external supervision will further strengthen the role of face by creating a potential constraint on farmers’ environmental damage.

**Figure 1:**
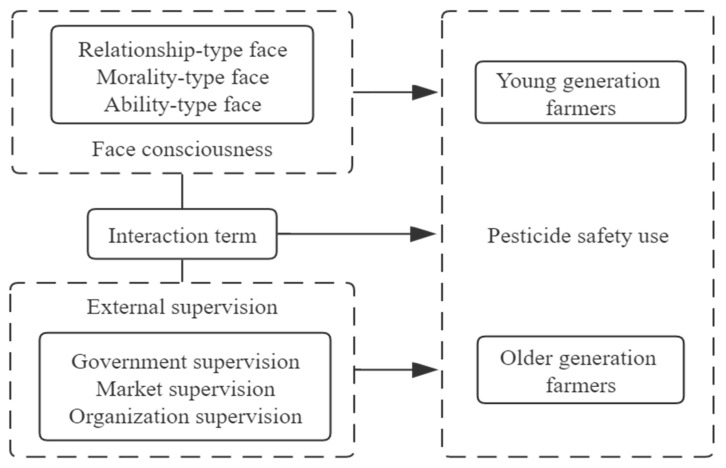
Analytical framework based on the impact of external supervision and face consciousness on pesticide safety use.

In addition, farmers of different generations are at different stages of their life cycle [44] and demonstrating distinct differences in growth experiences, subjective judgments, and behavioral logic; thus, their cognition and behaviors may also differ when faced with decisions about safe production. There is a relative lack of research on intergenerational differences in farmers’ pesticide safety use, so, to fill this gap, this study will explore the behavior of pesticide safety use from the perspective of various generational differences. Therefore, based on the above analysis, we draw the analytical framework diagram (see Figure 1) and propose the following hypotheses:

**Hypothesis** **1** **(H1).** *External supervision has a significant positive effect on farmers’ pesticide safety use.*

**Hypothesis** **2** **(H2).** *Face consciousness has a significant positive effect on farmers’ pesticide safety use.*

**Hypothesis** **3** **(H3).** *The impact of face consciousness and external supervision on farmers’ safe pesticide use has an interactive effect.*

## 3. Materials and Methods

### 3.1. Data Sources

The subjects of this study are citrus growers. Citrus is an important cash crop for billions of people in China, and, in terms of production, China’s citrus ranked first in the world in 2019. Meanwhile, as a crucial agricultural industry, it not only profoundly influences the development of China’s economy, but also has helped millions of farmers to get rid of poverty, which has an important social value. Moreover, as a perennial cash crop with stronger asset specificity and higher marketability, the study of pesticide use behavior of such farmers is representative. Sichuan is the key area for citrus production and agricultural technology promotion, with good demonstration and representation. To investigate the adoption status of green production technology, we conducted a field survey in rural areas of Sichuan from July to August 2020. The samples were selected by a combination of typical and random sampling, and the specific process was as follows: firstly, 10 sample counties (districts) were selected from areas with good citrus cultivation and economic development, covering areas in central, southeastern, and northeastern Sichuan, etc. Secondly, 2–3 sample townships were selected in each sampled county (district) based on information provided by the local agricultural bureau. Then, 3 sample villages were randomly selected from each sample township. Finally, this study randomly selected sample farmers from these sample villages to conduct “one-to-one” interview surveys. We issued totally 590 questionnaires. Subsequently, we screened the final collected data, eliminated blank questionnaires, carefully checked possible input and logical errors, and processed extreme values and outliers accordingly, resulting in a valid sample of 534, with an efficiency rate of 90.5%. The main contents included respondents’ characteristics, family and production characteristics, green production awareness and behavior, training, etc.

### 3.2. Basic Characteristics of Sample Farmers

From Table 1, the farmers were mainly middle-aged and elderly males with less than 9 years of education. The proportion of those aged 50 and above reached 70.0%; farmers with educational years at junior high school or below accounted for the largest proportion, at 83.7%; the planting area was mainly concentrated in less than 5 mu, accounting for 44.4%; and the total family population was mostly distributed between 3 and 4 persons, accounting for 44.0%, followed by 5 to 6 persons, accounting for 33.3%. In general, the sample farmers showed the essential characteristics of higher age, lower education level, smaller planting area, and larger population size, which is generally consistent with the actual situation in rural China.

### 3.3. Variable Selection

#### 3.3.1. Dependent Variable

Based on the prominent problem in green production practices—the proportion of safe pesticide use is generally low, considering the specific situation of pesticide safety use among farmers in the sample area, simultaneously, following the rigor of explanatory variables measurement, this study finally selects “whether farmers use pesticide safely” as the dependent variable, which is a dichotomous variable. The pesticide safety use means that farmers use pesticides in accordance with the “Guidelines for the Rational Use of Pesticides” issued by the General Administration of Quality Supervision, Inspection and Quarantine of the People’s Republic of China, and, if they answer yes, the variable will be assigned a value of 1, and, vice versa, a value of 0.

#### 3.3.2. Independent Variables

There are no rigorous criteria for measuring external supervision indicators in the existing literature. Combining the above analysis of external supervision and referring to existing research, this study selects government, market, and organization supervision to measure it at three levels: macro, medium, and micro. Among them, government supervision is measured by “the strictness of government supervision on green production behavior”; market supervision is measured by “market supervision mechanism has an impact on your green production behavior”; and organization supervision is measured by “the strictness of cooperative supervision on green production behavior.” The measurement option of market supervision is “strongly disagree” to “strongly agree”, and the remaining two are “strongly not strict” to “strongly strict”, and all three are assigned values from 1 to 5 in turn.

The core definition of face consciousness is “individual’s subjective perception of self-image and others’ recognition of the individual’s social image.” Therefore, according to the above correlation analysis, combined with the characteristics of Chinese native face culture and referring to the existing research [38,45], this study classifies face consciousness into three dimensions: ability-type face, relationship-type face, and morality-type face. Ability-type face is mainly reflected in an individual’s desire to show his or her ability; morality-type face focuses more on the moral psychology or moral (face) constraints that individuals have on their behavior; relationship-type face refers to individual’s tendency to build a good reputation and image in interpersonal interactions. Therefore, we use “adopting green production modes can reflect environmental ability”, “adopting non-green behaviors makes me feel guilty and guilty”, and “damage to the environment can affect reputation in social relations”. The options for the three are from “strongly disagree” to “strongly agree” and assigned values from 1 to 5, respectively.

#### 3.3.3. Control Variables and Instrumental Variable

It had been confirmed that personal and family characteristics were important factors influencing pesticide safety use among farm households [46,47,48], while, to further control for factors that may affect it among farmers, the study selects control variables in terms of both personal and family characteristics. Specifically, for personal characteristics, we introduce gender, age, years of education, whether a village cadre, and physical health status. For family characteristics, it presents “Do you have relatives and friends working in the government?”, the distance from your home to the nearest market, annual total family income, and village topography. Moreover, considering the possible endogeneity of the estimated results, the study selects “relational atmosphere (can you get along well with others?)” as an instrumental variable, which directly affects face consciousness to some extent. Still, it does not affect farmers’ pesticide safety use. Generally, it can only influence farmers’ behavior through their face consciousness. The results of definition and assignment of each variable are shown in Table 2.

### 3.4. Methods

This study focused on farmers’ pesticide safety use behavior. The research group understood farmers’ pesticide safety use by asking “Whether to use pesticides in accordance with safety standards”, which is a binary variable. Meanwhile, it has been found that personal characteristics affect the explanatory variables “linearly” in a linear model and “non-linearly” in a nonlinear model, resulting in a reflection problem (a technical difficulty in the identification of cohort effects). However, the reflection problem can be avoided in the binary logit model [49]. Therefore, this study employed a binary logit model for regression with the following equation:

(1) $$P_{i}=F\left(y\right)=\frac{1}{1+e^{-y}}\;$$

In Equation (1), $P_{i}$ represents the probability of pesticide safety use, and y represents farmers’ behavior. y=1 means that farmers have used pesticide safely; y=0 means that farmers have not used safely. y is the linear combination of variables X, W, and M, i.e.,:

(2) $$y=\alpha X+\beta W+\theta M+b_{0}$$

In Equation (2), X is the control variables, including householders’ personal and family characteristics; W is the external supervision, including government, market, and organization supervision; and M is face consciousness, including ability-type face, morality-type face, and relationship-type face; α, β, and θ denote the coefficients to be estimated; $b_{0}$ is the constant term.

By adequately processing Equations (1) and (2), the expression of the Binary Logit model can be obtained as follows:

(3) $$ln\frac{P_{i}}{1-P_{i}}=b_{0}+\alpha X+\beta W+\theta M+\varepsilon$$

In Equation (3), ε is a random error term. Some studies have suggested that neither the OLS model nor the Binary Logit model has an effect on the significance and direction of the coefficients of variables [50]. Based on this, this study uses the Binary Logit model for regression analysis along with robustness testing using the OLS model.

## 4. Results

### 4.1. Main Effects Analysis for External Supervision and Face Consciousness Affecting Farmers’ Safe Pesticide Use Behavior

From Table 3, the effects of government, market, and organization supervision on farmers’ pesticide safety use all pass the significance test with positive coefficients, showing that external supervision has a significantly positive effect on it. The stronger the external supervision, the greater the possibility of farmers using pesticides safely. The estimation results of OLS remain largely consistent with those of Binary Logit, suggesting that the model estimation results have strong robustness; therefore, hypothesis H1 is positively validated. Notably, the coefficient and significance of market supervision are 0.410 and 1%, respectively, which are bigger than those of government (0.318 and 5%) and organization supervision (0.225 and 10%), showing that market supervision has the most substantial effect on the use. Further, the marginal effects of government, market, and organization supervision on the use are 5.345%, 6.903%, and 3.789%, respectively. Other things being equal, the probability of farmers using pesticides safely increases by 5.345%, 6.903%, and 3.789% for each additional unit of these three types of supervision, respectively. In further analysis, the higher the stringency of government supervision, the more likely farmers are to use pesticide safely. This finding is consistent with previous studies [3,11].

In terms of face consciousness, both ability-type face and relationship-type face in the Logit model pass the significance test with positive coefficients, while the estimation results of the OLS model are generally consistent with them, but the morality-type face does not pass, demonstrating that ability-type face and relationship-type face can significantly and positively promote farmers to practice safe pesticide use, but morality-type face may not do so. That is, hypothesis H2 is partially positively tested. Further observation can reveal that the coefficient and significance of ability-type face are 0.395 and 5%, respectively, which are bigger than those of relationship-type face (0.315 and 10%), which shows that its role is relatively stronger in promoting farmers’ pesticide safety use compared to relationship-type face. Furthermore, there are marginal effects of 6.648% and 5.303% for the two types of face consciousness, respectively, i.e., for every 1 unit increase in these two types of face consciousness, the probability of farmers applying pesticides according to the standard increases by 6.648% and 5.303%, respectively, all other things being equal.

### 4.2. Endogenous Test of Main Effects

The above analysis has demonstrated the significant main effects. However, caution must be exercised in interpreting the effect of face consciousness on pesticide safety use. The reasons are as follows: to begin with, as farmers strictly comply with safe production standards, their face consciousness is enhanced accordingly, i.e., there is a causal relationship between the two; to follow, it is possible that both face consciousness and the use may be affected by unobserved omitted variables. Furthermore, possible measurement errors may also cause the estimation bias; thus, the results may have endogeneity issues. Based on the above analysis, the study refers to existing research [44] to construct a new dummy variable—“positive face consciousness” (Assign “positive face awareness” to 1 when all three proxy variables for face awareness are 4 or 5 at the same time; otherwise, assign it to 0), and also incorporates the instrument variable (Can you get along well with others?) as an endogenous variable of “positive face consciousness” in the IV-2SLS for a two-stage regression. The advantage of using IV-2SLS is that it overcomes the shortcomings of the ordinary least squares method for parameter estimation by systematically estimating face consciousness and pesticide safety behavior as interacting system endogenous variables. Moreover, IV-2SLS does not impose strict restrictions on the distribution of variables.

In Table 4, the regression results from the first stage show that relationship atmosphere has a significantly effect on farmers’ positive face consciousness, and the Wald endogeneity test results show that the hypothesis of no endogeneity is rejected at the 1% level. Additionally, the F-value of the joint significance test is 30.75, which is greater than the critical value of 16.38 at the 10% level [51], showing that it is appropriate to use relationship atmosphere as the instrumental variable for positive face consciousness, with no weak instrumental variable issue. In the estimated results of the second stage of IV-2SLS, the coefficient of positive face consciousness is 0.507 and passes significance test at the 5% level. It is further observed that the coefficients of estimated results also pass significance tests at the 5% and 1% levels in the Logit and OLS models, respectively, illustrating that the above regression results are reliable, and increasing positive face consciousness does improve the possibility of farmers using pesticides safely.

### 4.3. The Effect of Interaction Terms between External Supervision and Face Consciousness on Farmers’ Pesticide Safety Use

In fact, whether external supervision or face consciousness, the effects of both on farmers’ pesticide safety use are not independent and constant, but are in dynamic interaction changes; most likely there is an interaction effect. Therefore, the interaction terms of these two are constructed and included in the model for testing, and the estimation results are shown in Table 5.

According to Table 5, it is noteworthy that the interaction terms consisting of organization supervision and all dimensions of face consciousness fail the significance test. However, the interaction term between government supervision and morality-type face is significantly positive at the 1% level, demonstrating that it has a significantly positive effect on farmers’ pesticide safety use. Specifically, the effect of government supervision on farmers’ pesticide safety use is influenced, to some extent, by farmers’ morality-type face. In addition, explained in another way, the effect of farmers’ morality-type face on the use is also influenced by government supervision. Stronger government regulation will undoubtedly reduce the occurrence of moral hazard among farmers [52] and contribute to their safe production practices, while increased moral awareness among farmers will enhance this positive effect of government supervision. Next, the coefficient of interaction term between market supervision and ability-type face is significantly positive at the 5% level, showing a significant interaction effect of both on the use. The effect of market supervision on the use is influenced, to some extent, by ability-type face. Interpreted in another way, the effect of ability-type face on the use is also affected by market supervision. For farmers with higher ability-type face, the stronger the market supervision, the more it contributes to their safe pesticide use behavior. Farmers with stronger ability-type face, their own already sufficient endogenous motivation for safe pesticide application, and market supervision can further stimulate their motivation and strengthen the sustainability of their safe use. Finally, the interaction term between market supervision and relationship-type face is significantly positive at the 5% statistical level, which indicates that the higher the level of market supervision, the more likely farmers with high relational face are to use pesticide safely; in other words, the effect of market supervision on farmers’ use is influenced by the strength of relationship-type face. Hypothesis H3 is verified, i.e., there is an interactive effect of external supervision and face consciousness on farmers’ safe pesticide use, in that, while one side has a driving effect on the use, the other side can act as a facilitating mechanism to enhance this effect.

### 4.4. Analysis of Intergenerational Differences in the Effects of External Supervision and Face Consciousness on Pesticide Safety Use

Different generations of farmers have different values, perceptions, and behavioral choices [53]. For the current generational divisions, mainly 55, 60, and 65 years old [54], no fully uniform division has been developed, while 60 years old is less controversial. However, considering the lag of intergenerational differences on farmers’ awareness and behavior, together with the average age of sample farmers (55.056), this study finally extends forward 5 years from 60 years old and uses 55 years old as the division standard for group regression (older than 54 years old for the older generation farmers and younger than 55 years old for the young generation farmers). From Table 6, in terms of the mean values of pesticide safety use, external supervision, and face consciousness, the young generation farmers are greater than the older ones, indicating that the young have a better recognition and acceptance of pesticide safety use than the older, while they have a stronger perception of external supervision, and also a stronger face consciousness.

Table 7 reports the differences in the effects of external supervision and face consciousness on pesticide safety use between the older and young generations of farmers. Specifically, the effect of government supervision is significant at the 1% level for the young farmers, but not for the older. Further, organization supervision has a significantly positive effect on it for older farmers, but not for the young. Evidently, this suggests that government supervision may be more applicable to safe application behavior among young farmers, while organization supervision can be more effective in safe production behavior among the older. It is interesting to note that market supervision has a significantly positive effect by both the older and young farmers. It is not difficult to understand, whether young or older, economic interests are still their focus, and this is also an important manifestation of rational smallholder theory. Regarding face consciousness, the effect of ability-type face by the young generation farmers is significantly positive at the 1% level, showing that it can function positively to drive them into safe pesticide use, but the effect on the old ones is not significant. Moreover, the relationship-type face has a significantly positive effect on the older, but does not on the young.

## 5. Discussion

Empirical results above have shown that external supervision and face consciousness have significant and differential effects on pesticide safety use. With regard to external supervision, it has always been an essential external force in reducing farmers’ moral hazard [55]. In fact, enhanced penalty and strict regulations expose farmers to greater penalty costs if they are found to use prohibited pesticides or overuse pesticides; following the “rational economic man” perspective, it may be a more rational choice for them to comply with safe production standards to avoid additional penalty costs. A sound market selection mechanism allows for quality grading and products elimination, which means that farmers not participating in safe production will face serious excess profit losses, while the premium effect of quality agro-products from peers will compete for more profits. Forced by market competition, farmers will engage in safe pesticide use in pursuit of greater profits. Co-operatives rooted in rural communities have comparative advantages in monitoring farmers’ productive behaviors. For rural China, co-operatives are not only an economic organization, but also a village-level organization, which can rely on specific physical and institutional resources to develop certain supervisory effectiveness, which will be conducive to regulating farmers’ production behaviors. According to Table 3, among external supervision, market supervision has the highest effectiveness, which may be related to the characteristics of farmers’ pursuit of profit maximization, followed by governmental and organization supervision, respectively, where the government, as a formal organization capable of creating coercive power through administrative means, tends to be more effective in binding than co-operatives.

Viewed at the aspect of face consciousness, environmental protection certainly represents responsibility and competence in the context of sustainable development. Farmers are more willing to use pesticides safely when they perceive ecological conservation as a reflection of their competence and social responsibility. The finding resembled that of related studies. For example, pro-social behaviors, such as participation in environmental protection, could bring higher prestige to individuals, who were not only seen as trustworthy, but more popular in their circles [56], and such behaviors can even lead to an improvement in social status, and high social status means that individuals may have access to more resources and, thus, are more likely to engage in pro-environmental behavior [39]. The tight-knit network of relationships in rural China serves as an essential support for farmers to conduct production and lives. Currently, farmers no longer focus on a single economic interest; environmental values also influence their choice preferences [57,58]. Unsafe pesticide use may infringe on the ecological interests of other farmers, which, in turn, incur complaints and condemnation from others, resulting in damage to their reputation and image in rural social relations. Therefore, the stronger the relationship-type face of farmers, the more likely they are to apply pesticides safely. Unfortunately, the effect of morality-type face is not significant. Although, previous research has concluded that farmers’ moral awareness is closely related to their pro-environmental behavior [59]. However, moral identity theory suggests that individual characteristics are essential factors in the existence of strong and weak differences in moral identity. Table 1 demonstrates the characteristics of the sample farmers with high age and low education level; logically, their moral consciousness tends to be at low level, which renders morality-type face powerless to promote safe pesticide use among farmers.

We also performed regressions with intergenerational differences grouped and, according to Table 7, the safe use of young generation farmers is primarily influenced by government, market supervision, as well as ability-type face, while that of the older ones is mainly affected by organization, market supervision, and relationship-type face. The reason for this is that young generation farmers are more likely to understand and accept the purpose and content of government regulation. Further, they tend to be in their career period with larger family burdens, who need various administrative support in their later production, so they value government supervision more and, in fact, this phenomenon is also common in rural China. Most of the older farmers have lived in rural communities for quite some time, and co-operatives have more frequent contact with them, so it is easier to monitor their production behavior. In rural China, most young generation farmers have the outworking experience and are influenced by urban civilization, their ideology and adaptability are higher than those of the older generation, and they tend to see safe production more as competence and responsibilities, which is an important reason why they are willing to use pesticides safely. Compared to the young, the older have a deeper understanding of traditional Chinese social relationships and attach more importance to the relational face. In contrast, the young have been working outside far too long and have relatively weak knowledge of local relationships.

In future efforts to promote the safe use of pesticides, we especially unleash the full potential of market supervision, through the establishment and improvement of market selection mechanisms, to pressure farmers to use pesticides safely. “To forge iron, you need a strong hammer”, the government itself should fulfill supervisory responsibilities, implement regulatory measures, and moderately strengthen the supervision to safe production. Focusing on playing the auxiliary supervision role of peasant co-operatives, if necessary, the government can appropriately give co-operatives certain supervisory powers. It should capitalize on what the face consciousness of different age groups can do. Further, it should incorporate the ecological and collective significance of safe pesticide use actions into village bulletin boards to create an ecologically oriented culture, and establish a public announcement system for pesticide abuse to stimulate farmers’ face consciousness for safe production.

The limitations of this study are threefold. For one thing, the data used in the study do not reflect the dynamic effects of external supervision and face consciousness on farmers’ pesticide safety use behavior. Due to the difficulties in obtaining dynamic panel data on farmers’ production behavior, only static cross-sectional data can be used for the relevant analysis, resulting in the study’s findings failing to reflect the dynamic changes in farmers’ safe pesticide use behavior at varying stages of external supervision implementation, as well as the impact on farmers’ safe production at different periods of face consciousness changes. For another, all the samples of farmers used in this study are from Sichuan province, and the findings of the study cannot reflect the overall situation of the whole country; the agricultural production environment and face culture vary in different regions, and the differences in farmers’ production behavior cannot be reflected. Finally, in terms of sample size, due to time and cost constraints, a larger sample could not be tested to strengthen the generalizability of findings. To remedy these shortcomings, future studies will use richer panel data or national sampling data and expand the sample size to explore in depth the effects of external supervision and face consciousness on farmers’ pesticide safety use.

## 6. Conclusions

Using 534 farm household survey data from Sichuan province, China in 2020, this study empirically analyzed the effects of external supervision, face consciousness, and their interaction effects on farmers’ pesticide safety use, and conducted intergenerational differences analysis. The following conclusions were drawn.

Firstly, external supervision and face consciousness can effectively contribute to farmers’ pesticide safety use. However, among them, the effect of morality-type face is not significant. The marginal effects of government, market, and organization supervision are 5.345%, 6.903%, and 3.789%, respectively, and those of ability-type face and relationship-type face are 6.648% and 5.303%, respectively. Secondly, there is an interactive effect between external supervision and face consciousness on pesticide safety use. That is, where one party acts on the safe use of pesticides by farmers, the other party is able to serve as a moderating mechanism to enhance this effect. Finally, there are significant intergenerational differences in the effects. The behavior of young generation farmers is influenced by government, market supervision, and ability-type face, while that of older generation farmers is mainly affected by market, organization supervision, and relationship-based face.

## Figures and Tables

**Table 1:** Basic characteristics of sample farmers.

Variables	Classification	Sample Size	Proportion/%
Gender	male	381	71.3
	female	153	28.7
Age/years old	<50	160	30.0
	50~65	289	54.1
	>65	85	15.9
Educational years	≤6	232	43.4
	7~9	215	40.3
	≥10	87	16.3
Planting area/mu	<5	237	44.4
	5~10	127	23.8
	10~20	81	15.2
	≥20	89	16.6
Total family population/person	≤2	86	16.1
	3~4	235	44.0
	5~6	176	33.0
	≥7	37	6.9

Notes: 1 mu is approximately equal to 666.67 square meters.

**Table 2:** Variable definition and descriptive statistics.

Variable Definition	Assignment	Mean	Std. Dev
Whether to use pesticides in accordance with safety standards	Yes = 1, No = 0	0.434	0.496
Strictness degree of government supervision on green production behavior	strongly not strict = 1, not strict = 2, general = 3, strict = 4, strongly strict = 5	3.676	1.086
Market supervision mechanism has an impact on your green production behavior	strongly disagree = 1, disagree = 2, general = 3, agree = 4, strongly agree = 5	3.264	1.071
Strictness degree of cooperative supervision on green production behavior	strongly not strict = 1, not strict = 2, general = 3, strict = 4, strongly strict = 5	3.056	1.098
For environmental protection, you hope others can identify you with green production mode	strongly disagree = 1, disagree = 2, general = 3, agree = 4, strongly agree = 5	3.921	0.761
You will feel shame and guilt without green production mode	strongly disagree = 1, disagree = 2, general = 3, agree = 4, strongly agree = 5	3.438	1.043
Behaviors of destroying ecological environment will affect own reputation and be condemned by others	strongly disagree = 1, disagree = 2, general = 3, agree = 4, strongly agree = 5	3.305	1.060
Gender	female = 0, male = 1	0.713	0.453
Age	actual age of interviewees	55.056	10.042
educated years	years of education/year	7.525	3.532
Are you a village cadre?	Yes = 1, No = 0	0.097	0.297
How is your health?	very poor = 1, poor = 2, general = 3, good = 4, very good = 5	3.930	0.790
Do you have relatives or friends working in the government departments?	Yes = 1, No = 0	0.182	0.386
The distance from your home to the nearest market	actual distance/km	3.708	3.111
Annual total family income	annual household income in 2019/ten thousand	2.608	1.145
Village topography	plain = 1, hill = 2, mountain = 3	2.022	0.401
Can you get along well with others?	Yes = 1, No = 0	0.629	0.483

Notes: To avoid the influence of heteroscedasticity, we take the logarithm of the variable of household annual total income.

**Table 3:** Model estimation results of external supervision and face consciousness on farmers’ pesticide safety use.

Variable Name	Logit		OLS	
	Coefficient	Marginal Effect (%)	Coefficient	Marginal Effect (%)
Government supervision	0.318 ** (0.129)	5.345	0.053 ** (0.022)	5.256
Market supervision	0.410 *** (0.154)	6.903	0.076 *** (0.027)	7.639
Organization supervision	0.225 * (0.135)	3.789	0.043 * (0.025)	4.230
Ability-type face	0.395 ** (0.183)	6.648	0.065 ** (0.030)	6.518
Morality-type face	0.109 (0.158)	1.833	0.015 (0.028)	1.487
Relationship-type face	0.315 * (0.172)	5.303	0.061 ** (0.031)	6.087
Gender	−0.303 (0.244)	−5.092	−0.052 (0.042)	−5.217
Age	0.003 (0.013)	0.056	0.001 (0.002)	0.061
Years of education	0.135 *** (0.040)	2.278	0.023 *** (0.007)	2.280
Whether village cadre	−0.313 (0.378)	−5.264	−0.043 (0.066)	−4.327
Health status	−0.257 * (0.152)	−4.319	−0.040 (0.025)	−3.983
Do you have relatives and friends working in the government?	0.218 (0.283)	3.670	0.035 (0.049)	3.517
The distance from your home to the nearest market	0.052 (0.036)	0.870	0.011 * (0.006)	1.110
Annual total family income	0.038 (0.105)	0.647	0.006 (0.018)	0.584
Village topography	0.014 (0.283)	0.231	0.004 (0.048)	0.384
Pseudo R^2^	0.259	—	—	—
Adjusted R^2^	—	—	0.284	—

Notes: ***, **, and * represent the significance level at 1%, 5%, and 10%, respectively; standard error in parentheses.

**Table 4:** The effect of positive face consciousness on farmers’ pesticide safety use.

Variables	2SLS		Logit	OLS
	First-Stage	Second-Stage		
Relationship atmosphere	0.196 *** (0.035)	—	—	—
Positive face consciousness	—	0.507 ** (0.212)	0.547 ** (0.250)	0.121 *** (0.048)
Other variables	Controlled			
F-Value	30.75	—		
Pseudo R^2^/Adjusted R^2^	—		0.244	0.275

Notes: The dependent variable in the first stage of the regression results is positive face consciousness, and the dependent variable in the second stage is pesticide safety use; *** and ** indicate significance at the 1% and 5% statistical levels, respectively, with standard errors in parentheses.

**Table 5:** The effect of the interaction terms of external-supervision- and face-consciousness-related variables on pesticide safety use.

Variables	Logit		OLS	
	Coefficient	Standard Error	Coefficient	Standard Error
Government supervision * Morality-type face	0.585 ***	0.202	0.096 ***	0.032
Market supervision * Ability-type face	0.626 **	0.250	0.088 **	0.037
Market supervision * Relationship-type face	0.472 **	0.237	0.083 **	0.036
Control variables	Controlled			
Pseudo R^2^	0.291	—	—	—
Adjusted R^2^	—	—	0.306	—

Notes: Some of the results with insignificant effects are not presented due to space limitations, other variables are consistent with Table 2, and the estimation results are omitted; ***, **, and * indicate significance at the 1%, 5%, and 10% statistical levels, respectively; to avoid the interference of multicollinearity, the variables are decentered before the interaction terms are performed.

**Table 6:** Descriptive statistics of selected variables for both old and new generations of farmers.

Variables	Young Generation Farmers		Older Generation Farmers	
	Mean	Std. Dev	Mean	Std. Dev
Pesticide safety use	0.520	0.501	0.362	0.481
Government supervision	3.803	1.078	3.569	1.083
Market supervision	3.484	1.012	3.079	1.087
Organization supervision	3.250	1.111	2.893	1.062
Ability-type face	4.094	0.693	3.776	0.786
Morality-type face	3.656	1.012	3.255	1.034
Relationship-type face	3.557	1.019	3.093	1.050

**Table 7:** Intergenerational differences in regression results for the effects of external supervision and face consciousness on pesticide safety use.

Variables	Young Generation Farmers		Older Generation Farmers	
	Logit	OLS	Logit	OLS
Government supervision	0.440 ** (0.177)	0.091 *** (0.034)	0.115 (0.206)	0.010 0.030
Market supervision	0.389 * (0.216)	0.073 * (0.042)	0.512 ** (0.244)	0.080 ** 0.035
Organization supervision	0.050 (0.199)	0.005 (0.038)	0.554 *** (0.208)	0.091 *** 0.033
Ability-type face	0.662 ** (0.276)	0.122 ** (0.051)	0.166 (0.264)	0.033 0.038
Morality-type face	0.097 (0.223)	0.023 (0.042)	0.060 (0.269)	0.004 0.037
Relationship-type face	0.205 0.252	0.043 (0.047)	0.461 * (0.272)	0.070 * 0.042
Pseudo R^2^	0.214	—	0.340	—
Adjusted R^2^	—	0.214	—	0.337

Notes: Some of the results with insignificant effects are not presented due to space limitations; ***, **, and * represent the significance level at 1%, 5%, and 10%, respectively; standard error in parentheses.

## Data Availability

The data presented in this study are available within the article.
